# Acceptability and feasibility of tuberculosis-diabetes mellitus screening implementation in private primary care clinics in Yogyakarta, Indonesia: a qualitative study

**DOI:** 10.1186/s12889-023-16840-z

**Published:** 2023-10-03

**Authors:** Denny Anggoro Prakoso, Wahyudi Istiono, Yodi Mahendradhata, Merita Arini

**Affiliations:** 1 Doctoral Program of Medical and Health Sciences, Faculty of Medicine, Public Health and Nursing, Universitas Gadjah Mada, Yogyakarta, Indonesia; 2 Department of Family and Community Medicine, Faculty of Medicine, Public Health and Nursing, Universitas Gadjah Mada, Yogyakarta, Indonesia; 3 Department of Health Policy and Management, Faculty of Medicine, Public Health and Nursing, Universitas Gadjah Mada, Yogyakarta, Indonesia; 4 Master of Hospital Administration, Postgraduate Program, Universitas Muhammadiyah Yogyakarta, Yogyakarta, Indonesia

**Keywords:** Acceptability, Feasibility, Tuberculosis, Diabetes Mellitus, Screening, Primary care

## Abstract

**Background:**

The relationship between Tuberculosis (TB) and Diabetes Mellitus (DM) is intricate and intertwined, posing significant global health challenges. In addition, the increasing prevalence of DM worldwide raises concerns regarding the potential resurgence of tuberculosis. The implementation of tuberculosis prevention strategies is of the utmost importance, especially in countries like Indonesia that encounter a dual burden of TB and DM. The significance of TB screening in private primary care settings for patients with diabetes cannot be overstated. Implementing TB screening protocols in private primary care settings can assist in identifying diabetic patients with tuberculosis. Therefore, this study aims to explore the acceptability and feasibility of tuberculosis-diabetes mellitus screening implementation in private primary care clinics.

**Methods:**

We conducted implementation research with an exploratory qualitative design. Fifteen healthcare professionals from five private primary health care clinics in Yogyakarta, Indonesia, participated in five focus groups. The discussions were audio recorded, transcribed verbatim, and thematically analyzed. As part of the feasibility assessment, surveys were conducted in each clinic. We conducted a thematic analysis in accordance with the theoretical framework of acceptability and the feasibility assessment.

**Results:**

We identified that most private primary care clinics deemed the implementation of TB screening in DM patients acceptable and practicable. We revealed that the majority of diabetes patients enthusiastically accepted TB-DM screening services. In addition, we found that the healthcare professionals at the clinic are aware of the nature of the intervention and demonstrates a positive attitude despite a subtle burden. The stigma associated with COVID-19 has emerged as a new implementation barrier, joining TB stigma, lack of resources, and regulatory issues. We identify concealed and tiered screening as a potential method for enhancing the implementation of TB-DM screening.

**Conclusions:**

The implementation of TB screening in DM patients in private primary care clinics had the potential to be acceptable and feasible. To achieve a successful implementation, consideration should be given to supporting factors, hindering factors, and strategies to improve TB screening in DM patients.

## Introduction

Tuberculosis (TB) is a significant global public health problem. Despite efforts to control the disease, it remains a leading cause of death worldwide. According to the Global TB Report 2022, there were an estimated 10 million cases of TB worldwide in 2020, with 5.8 million cases among men and 3.8 million cases among women. TB burden is not evenly distributed worldwide, with the majority of TB cases occurring in low- and middle-income countries. In 2021, the 30 countries with high TB burden accounted for 80% of the global TB burden, with India, China, and Indonesia having the highest number of TB cases [1]. The report highlights that TB incidence has declined by 1.5% yearly since 2015, and TB mortality has decreased by 11% since 2010. However, progress in reducing TB incidence and mortality has been slow, with only a 5.6% reduction in TB incidence and a 9% reduction in TB mortality between 2015 and 2021 [1]. The COVID-19 pandemic has significantly impacted TB control, with disruptions in TB diagnosis and treatment services leading to a potential increase in TB-related deaths [1].

WHO regions in Southeast Asia (44%), Africa (25%), and the West Pacific (18%) have the most TB cases. Indonesia is a country that accounts for two-thirds of the global total of TB sufferers reaching 8.5% [2]. Indonesia ranks second with the highest TB burden in the world. It has the third-highest gap between the estimated incident cases and new case notifications [1]. About 30% of active TB cases are currently not detected by Indonesian health services, and approximately 44% of detected TB cases are not reported [3].

Data from the Indonesian TB Dashboard indicates 568.987 diagnosed TB patients in 2019. This number has decreased to 393.323 (in 2020) and 385.295 (in 2021). This condition may be related to the COVID-19 pandemic in early 2020 [4]. In fact, TB cases are estimated to have increased by 824,000 in 2021. It shows the government must exert greater effort to improve health services, especially TB screening and treatment [5].

Based on epidemiological data, apart from being classified as a country with a high pulmonary TB burden, Indonesia is also one of the ten countries with the highest incidence of Diabetes Mellitus (DM) worldwide. Indonesia is ranked 7th in the world with the number of DM sufferers, which reached 10.7 million in 2019 and is expected to increase to 13.7 million in 2030 [6]. The recent increase in DM cases, especially in countries where TB is also endemic, has led to a reemergence of the importance of DM as a risk factor for TB. DM-induced immunopathies result in decreased immunity that favors TB development, which may lead to a higher bacterial load [7–9]. There is an urgent need to implement TB prevention strategies among the millions of Mtb-exposed DM patients worldwide [10]. By combining the slower-than-expected decline in TB rates worldwide and increasing DM rates, the convergence of these two epidemics could lead to a resurgence of TB disease, especially in low- and middle-income countries [11–14].

The National Guidelines for Medical Services for the Management of Tuberculosis in Indonesia in 2020 mentioned that TB-DM comorbidity is included as a special condition that needs attention. Every DM patient must be screened for TB by examining TB symptoms and chest X-ray, whereas TB patients are screened for DM by checking blood glucose [15]. Some evidence has shown that screening for active TB in people with DM can accelerate case detection, resulting in earlier treatment and prevention of transmission. Giving TB preventive therapy to people infected with TB and DM can prevent the progression of TB. On the other hand, DM screening in individuals with TB can improve case diagnosis, early treatment, and tertiary prevention of DM. It can indirectly improve TB-specific treatment outcomes [16]. However, the implementation of this policy has not been fully absorbed and implemented routinely and systematically by all parties involved, especially in private primary health services.

In Indonesia, tuberculosis services are programmatically administered, with primary community health services (Puskesmas/Community Health Centers [CHCs]) functioning as the backbone of the National Tuberculosis Programs (NTPs) [17]. Private primary care providers (PPCs) have yet to play a significant role in tuberculosis management in Indonesia, despite the country’s health care system being sustained by a very large private sector [18]. A majority of patients favor private medical care. In spite of this, the private sector frequently fails to provide TB services with adequate quality [19]. In addition, PPCs have a tendency to report TB cases tardy and provide subpar services [20–22]. The private health sector is a major provider of health services and is considered a more accessible, responsive, and individualized option for patients. Failure to involve private service providers utilized by suspected and diagnosed TB patients hinders case detection, causes diagnostic delays, contributes to inadequate and ineffective treatment, heightens drug resistance, and imposes an unnecessary financial burden on patients [23].

Implementation of the outcome measurements is crucial for monitoring and assessing the success of new implementation efforts. Acceptability and feasibility are two critical implementation outcome indicators that reflect the program’s success in terms of stakeholder satisfaction and implementation in the real world. Both serve as indicators of the effects of implementation processes and as prerequisites for achieving the intended service delivery and clinical outcomes. Thus, the success of this screening implementation depends on its acceptability and feasibility [24, 25]. Due to the limited number of TB patients in private primary clinics, the context in this study was limited to one-way screening, namely TB screening in DM patients (TB-DM screening). Moreover, the implementation was conducted while the COVID-19 pandemic occurred. Consequently, this circumstance might have an impact on the implementation of research. This study aims to explore the prospective acceptability and feasibility of TB screening among DM patients in private primary care clinics in Yogyakarta, Indonesia.

## Methods

We conducted an exploratory study using a qualitative design. The exploratory studies approach is a dynamic and intellectually stimulating research method that provides the opportunity to venture into uncharted research territory, challenge assumptions, and contribute to the advancement of scientific knowledge through inductive exploration. This methodology emphasizes adaptability, openness, and the pursuit of new insights [26]. This study collects qualitative data through the use of focus groups. Focus groups are group interviews used to explore the expertise and experiences of participants, including how and why people conduct in particular ways [27]. Focus groups are useful for bringing together homogeneous groups of participants with pertinent expertise and experience on a particular topic so that they can share in-depth information [28]. This study aims to explore and describe the acceptability and feasibility of TB-DM screening implementation using the theoretical framework of acceptability (TFA) and the feasibility assessment [29, 30]. To ensure report quality, the 32-item Consolidated Criteria for Reporting Qualitative Research (COREQ) was applied [31]. The research team was comprised of DAP (1st author), a Ph.D. candidate in implementation research with an interest in tuberculosis research. WI (2nd author) is a Ph.D. expert in primary care and family medicine, YM (3rd author) is a Ph.D. expert in research implementation and public health, and MA (4th author) is a Ph.D. in health service research. DAP and MA worked at universities affiliated with private primary care clinics.

### Study setting

This study was conducted in Yogyakarta, a special province In Indonesia. We participated in a faith-affiliated private primary healthcare (Muhammadiyah clinics), Indonesia’s biggest private healthcare network, from October 2021 to December 2021. Five private primary health care in Yogyakarta were involved, including Clinic A, Clinic B, Clinic C, Clinic D, and Clinic E *(The clinic name was withheld for ethical reasons).* The five clinics were selected to represent the diversity of urban-rural clinics and inpatient-ambulatory care settings. Moreover, all clinics collaborated with the Indonesian Social Security Organizing Agency (BPJS-Kesehatan Indonesia), which provided health financing for public health services. None of the clinics offered radiology services or tuberculin testing. In the context of our study, these clinics have limited capabilities and can only provide symptom screening, sputum collection, and referrals of tuberculosis-suspected individuals to health facilities that offer services for diagnosis confirmation (sputum microscopy or geneXpert MTB/RIF examination). At the time the study was conducted, all of these clinics were affected by the COVID-19 pandemic. This condition has resulted in decreased clinic visits by diabetic patients compared to before the pandemic. This circumstance might indicate an external factor affecting the implementation’s success.

### Participants recruitment

We employed purposive sampling for identifying and selecting informants based on the characteristics of the sources of information/data required. Clinic directors, program managers and healthcare workers implementing TB-DM screening at each clinic serve as informants. They were selected to represent clinic stakeholders, administrators of TB-DM screening and healthcare workers who perform TB-DM screening examinations. The characteristics of the informants (15 people) are listed in Table 1. The eligibility requirements for the informant were active employment at the clinic and absence from paid leave. Before seeking approval, we contacted selected informants and provided information about the study background, procedures and methods.

### Data collection

Study evaluations were performed one month after the TB-DM screening had been implemented. Focus group discussions (FGDs) were conducted to assess the acceptability and feasibility of the implementation’s success indicators. FGDs were carried out from November to December 2021 by the first author (DAP) and fourth author (MA), who have been formally trained in qualitative research methods and were carried out under the supervision of qualitative research experts (YM, WI). The FGDs were conducted using a semi-structured interview guide developed and consulted with qualitative research experts and piloted with private clinics outside the five selected clinics. Questions in the FGD guide explored knowledge and experiences about the implementation strategy. The research team collected FGD data from each clinic at different times. Minimum of three participants attended each FGD. FGDs were held in meeting rooms at each clinic. Five FGDs were conducted in *Bahasa Indonesia* and took 60–90 min to complete. Each FGD interview was audio recorded.

FGD data collection was discontinued when data saturation was reached. Data saturation refers to the extent to which new data repeat what has been conveyed in previous data [32]. It indicated that participants reiterated the same answers or viewpoints and that no new information or perspectives emerged from the discussion [33]. The themes and ideas had been discussed, and the accumulation of additional data yielded no new insights. There were no additional interviews for data collection.

An organizational-level survey was conducted in each clinic as part of the feasibility assessment utilizing the feasibility of intervention measures (FIM). The instrument was completed by a team of healthcare professionals at each clinic based on an agreement reached during the FGD. FIM is one of three instruments that measure implementation outcomes, along with the Acceptability of Intervention Measure (AIM) and the Intervention Appropriateness Measure (IAM), and is considered one of the “key indicators” of successful implementation [34]. Service providers can use this instrument to assess the acceptability, appropriateness, and feasibility of a specific implementation strategy. These measures may be used separately or in combination, depending on the general instruction. We employ independent phases of feasibility assessment to evaluate practicality. According to the guidelines, the phases of feasibility assessment are designed pragmatically. This instrument’s administration, scoring, and interpretation do not require specialized training. There is currently no score cutoff available for interpretation, but higher scores indicate greater feasibility. According to the study’s findings, this FIM instrument consists of four valid and reliable implementation feasibility outcome measures. The AIM, IAM, and FIM demonstrated excellent psychometric properties in a series of studies. Specifically, the measures demonstrated content validity, discriminant content validity, reliability, structural validity, structural invariance, known-group validity, and change responsiveness. The predictive validity of the measures is currently being evaluated [24].

### Data analysis

Each FGD was transcribed verbatim by a researcher promptly following data collection. NVivo 12 + was used to manage and code all transcripts, contributing to the study’s reliability and validity. In addition, DA and MA ensured the integrity of the data by rechecking before and during coding. DA performed thematic data coding to obtain selective and axial coding and themes [35]. The analysis for the focus group discussion was conducted using the procedures outlined below: Initially, the discussion transcripts were examined in depth. Reading and rereading allowed the study to become familiar with the data and grasp the bigger picture. Second, initial codes were generated inductively for each discussion and compared to other discussions’ codes to identify recurring codes. Thirdly, essential groups/categories of the identified codes were established, and a specific theme was assigned to each group. DA and MA evaluated and discussed the codes and themes throughout the analysis. In addition, continuous data analysis was performed following each FGD session. A saturation evaluation was performed in which data collection ceased once data saturation was reached during analysis and no new categories were identified.

We conducted a thematic analysis with relevant construct guidelines from TFA, including intervention coherence, ethics, burden, and affective attitude, as well as feasibility instrument measurement outcome. In this study, thematic analysis was conducted by using data recognition and data coding to identify relevant segments, generating initial themes from coded segments, reviewing and refining these themes, defining and naming each theme, and analyzing and interpreting themes related to the research question theme. This analysis consisted of iterative procedures. To ensure trustworthiness, we utilized triangulation, debriefing, and member checking [36]. To help reconcile divergent opinions among analysts regarding the qualitative data analysis, we facilitated communication and collaboration, employed triangulation, and utilized an iterative approach.

### Ethics

Research ethics approval was acquired from the Research Ethics Committee of Universitas ‘Aisyiah Yogyakarta, Indonesia (Ref no: 1905/KEP-UNISA/XI/2021). The study was explained to participants using an informed consent form. Participation and recording of interviews were voluntary. Permission was obtained through a written consent form. To preserve confidentiality, the participant’s actual name and the clinic’s name were omitted from the report. Clinic directors and healthcare workers from each clinic were invited to participate in a focus group discussion, and all consented (100%).

## Result

We conducted interviews with FGDs involving 15 informants (Table 1), consisting of 9 general practitioners, 5 midwives and 1 nurse. Informants comprised males (n = 2) and females (n = 13) from five private primary care clinics. Five groups of healthcare professionals agreed and consented to engage in a group discussion as part of the data collection.

Some of the midwives who participated in this research were TB clinic administrators or program managers. Due to a personnel shortage, clinic healthcare professionals are required to multitask. This is a common occurrence in Indonesian private health services with limited human resources.

Laboratory personnel were unavailable at all clinics where we conducted our study. Most private primary care clinics have numerous limitations, such as a lack of a laboratory and human resources, which prompted part of our research.

All participants in the study provided only clinical services. None of them had been assigned TB surveillance duties. Each clinic is only compelled to report case findings on a routine basis to the national TB information system.

**Fig. 1:**
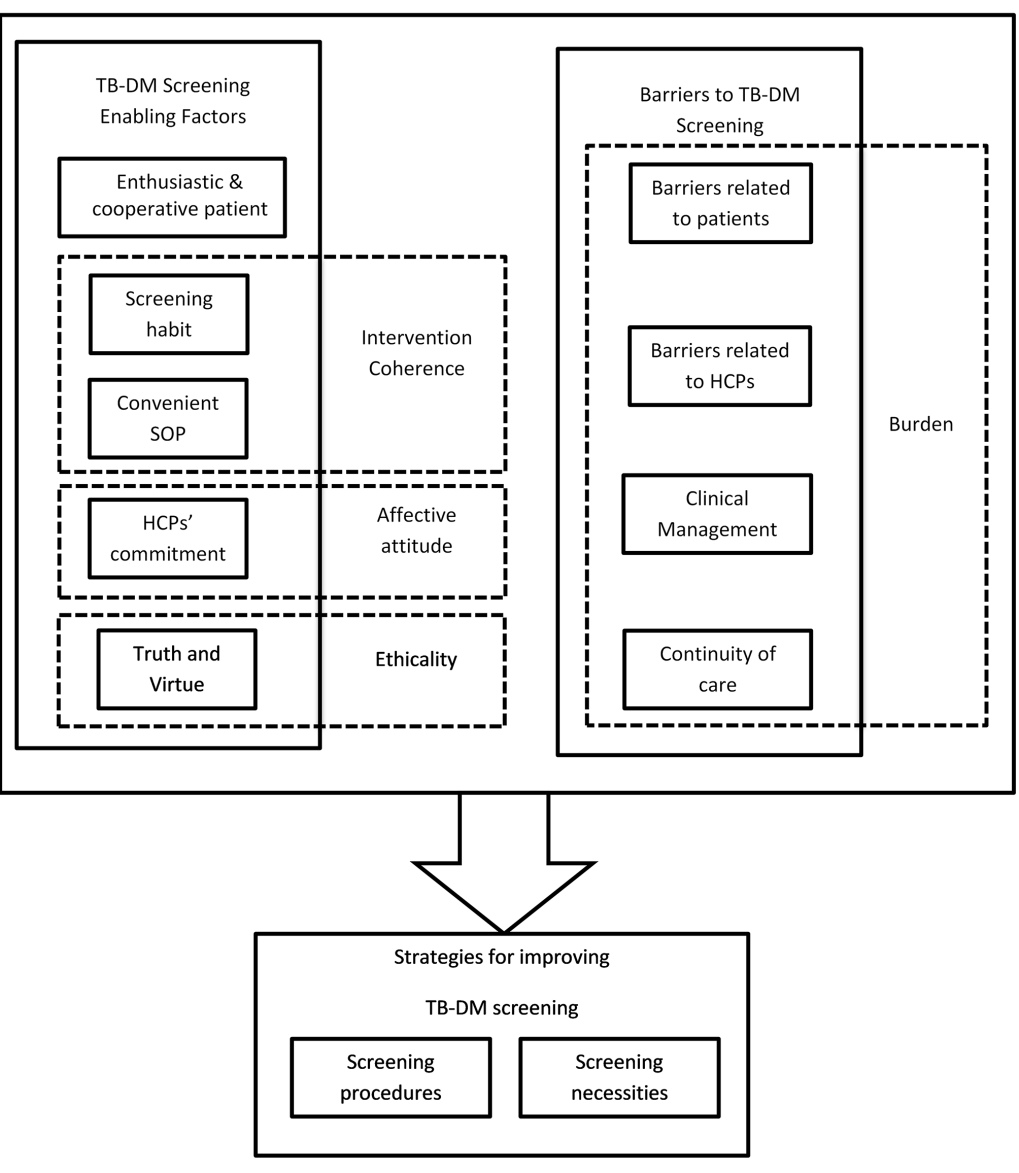
Themes and categories from focus group discussions

Based on the analysis of qualitative data obtained from informants, we found three themes developed from the findings (Fig. 1). The three themes related to (1) TB-DM screening enabling factors, (2) Barriers to TB-DM screening, and (3) Strategies for improving TB-DM screening. Our FGD results illustrated the acceptability and feasibility of TB screening among DM patients based on the TFA constructs and the feasibility framework. We adjusted and matched the FGD results’ data to the acceptability constructs. We set three constructs of acceptability (affective attitude, intervention coherence, ethicality) under TB-DM screening enabling factors. Meanwhile, the last construct (burden) was under the theme of barriers to TB-DM screening. Informant quotations from FGD were identified by informant code.

**Table 1:** Informants’ characteristics (n = 15)

Informant code	Age (year)	Gender	Position	Clinic
I1	37	F	Healthcare worker	A
I2	32	F	Program manager	A
I3	32	F	Clinic director	A
I4	31	F	Program manager	B
I5	26	M	Healthcare worker	B
I6	26	F	Clinic director	B
I7	26	F	Clinic director	C
I8	25	F	Program manager	C
I9	26	F	Healthcare worker	C
I10	43	F	Clinic director	D
I11	27	F	Program manager	D
I12	30	F	Healthcare worker	D
I13	32	M	Clinic director	E
I14	41	F	Program manager	E
I15	30	F	Healthcare worker	E

I = Informant; F = Female; M = Male

### Theme 1: screening enabling factors

We identified conditions that facilitated TB-DM screening in clinics. These factors included the commitment to support from clinic directors, clinic healthcare professionals and DM patients who were enthusiastic and cooperative. The enabling factors for implementing TB screening in DM patients were represented in interview excerpts.

*“… the implementation of TB screening in cooperative patients has no problems, then the clinical health workers have no objections (with the service), and the (clinic) leader also shows support.” (I10)*.

*“I feel that patients may wonder why I am being asked about complaints (TB symptoms), but from their facial expressions, they look happy, as if the health workers are paying more attention (their health).” (I11)*.

We identified the affective attitude construct that provided insightful information about how individuals perceived and engaged with the intervention. Affective attitude reflects the subjective experiences and emotional reactions of the clinic’s health care professionals towards the implemented intervention. We revealed that most participants found the intervention agreeable, appealing, and gratifying, indicating positive affective attitudes.

FGD participants discussed the evaluation of the implementation of TB screening in DM patients in their respective clinics. They found that clinic healthcare professionals generally accepted TB screening in DM patients. This study demonstrated that most clinic directors and healthcare professionals felt that TB screening could support clinical performance in providing documents for clinical accreditation purposes. In addition, they felt that it could increase patient perceived satisfaction and identify new cases of TB. Some clinics have committed to providing TB-DM treatment and screening services. Excerpts from interviews reflected support for implementation acceptance.

*“It is possible (with the implementation of TB screening) these results (data from screening result) can be used for preparation for clinic accreditation, used as quality indicators of TB program.” (I11)*.

*“Related to TB screening in DM patients, after we do the screening, it turns out that TB disease has started to appear in DM patients. Yes, this is very important to expand our clinical services.” (I4)*.

The acceptability of the intervention coherence construct was determined using participant data. This construct measured the extent to which clinical healthcare professionals comprehended the intervention and its mechanism of action. We noticed how well participants comprehended the intervention’s rationale, objectives, and procedures.

Furthermore, this study found that TB screening in DM patients was not a novel service activity; rather, it was viewed as an additional service and used to support continuous care planning efforts for their patients. The majority expressed an intention to undertake screening. They contended the Standard Operating Procedure (SOP) for TB screening in DM patients was identical to the SOP for other disease screening services, making TB-DM screening services simpler to administer. Interview excerpts illustrate the deployment of TB screening in accordance with the existing chronic disease services at each clinic.

*“Yes, the procedure of the TB-DM screening (SOP) is the same as (SOP) for other (chronic disease) patients… the difference is that the TB-DM screening is even more in-depth…not much different from our other services…”(I14)*.

*“…so with this TB-DM screening, we can know as early as possible whether the patient has TB or not, if for example (the patient) has been detected (the suspected patient) is immediately referred to the Health Center, later he can confirm positive or negative TB, (if positive) then there will be (plan) routine treatment.” (I8)*.

We determined ethical constructs of acceptability based on the data collected. We evaluated the extent to which an intervention or implementation strategy was morally acceptable, ethically competent, and consistent with the values, principles, and ethical standards of the clinic’s healthcare professionals. We observed that the majority of these implementations were ethically solid.

In general, according to clinical healthcare professionals, the implementation of TB screening in DM patients aligns with their expectations. Implementation procedures were consistent with the real conditions and virtue. In this case, there was no conflict with ethics, both in professional ethics and community traditions. Excerpts from interviews reflect the implementation of TB screening in DM patients and ethics.

*“In my opinion, the TB-DM screening is in line with expectations. TB-DM service is meant as an entry point for more holistic patient care. Indeed, we should explore the basic problem more thoroughly. If a DM patient has had a cough for 2 weeks, we suspect TB and check the sputum…”(I13)*.

*“This additional service for implementing TB screening seems good. It feels like a health worker is happy to be able to help patients if it turns out they have TB…”(I7)*.

### Theme 2: barriers to TB-DM screening

Barriers to implementing TB screening in DM patients were identified as patient-related barriers, healthcare professional-related barriers, clinical management, and continuity of care. The DM patients showed an impression of rejection due to their fear of the TB stigma and the emergence of the COVID stigma during the COVID-19 pandemic. Excerpts from interviews reflect patient-related barriers.

*“….So I have said that my patient will be screened for TB, but the patient looks scared. It seems that I am suspected of this (suffering from TB)… The patient becomes even speechless as if she doesn’t want to answer (the next question), sometimes the patient also (trying to) covers up illness…” (I14)*.

*“…Regarding the current COVID pandemic, when my patients ask about the condition of fever, cough, runny nose, or shortness of breath related to the complaint, they definitely say there isn’t any. They look scared and in denial because if they cough, they think they will definitely be “COVIDED” and later must be isolated.” (I5)*.

Additionally, several other factors caused patients to feel unwilling to receive TB screenings for various reasons, including the fact that they were asymptomatic, busy, and inclined to assume they felt certain they did not have TB. Several patients objected to being referred for the same reason based on the screening results of patients with suspected TB and referral indications.

*“…When asked about the symptoms of TB, the patient immediately makes the assumption (by himself) that he has no symptoms of TB and is unlikely to (suffer from) TB. For example, when I ask, “do you have a persistent cough and weight loss?“ The patient answered, “I am healthy, I don’t cough, I am not emaciated, I am fat, my environment is good…“(I1)*.

*“…(patients) when asked to tell (answer screening questions) there are (various) reasons, ‘oh I’m in a hurry, ‘I don’t have time, ‘oh dear, I’m busy, ‘I don’t have time for that’…”(I3)*.

The findings of our data analysis demonstrated the challenges encountered by healthcare workers, particularly those who did not conduct TB-DM screening due to excessive workload, patient queues, a lack of health workers in clinics, and gender roles. The quote about provider-related barriers reflects barriers from the staff’s point of view.

*“…the weakness we face in TB-DM screening is that sometimes we forget, because of other clinical work that makes us less concerned and not too focused on services (TB-DM screening)…” (I13)*.

*“…at that time, I forgot about the screening because there were so many queues of patients, it was chaotic, and we immediately got dizzy…“(I14)*.

Related to the TB-DM screening which was carried out in each clinic, based on the results of the discussion, we found several obstacles encountered related to clinical management, namely the screening instrument has not been integrated into the medical record, the weak reminder system, the lack of TB-DM education, afraid of increasing referral rates, lack of supporting facilities such as sputum rooms. The following excerpt shows clinical management-related barriers.

*“…regarding the TB screening sheet, the sheet is separate, we take it when there is a DM patient, we haven’t included the sheet into the medical record…” (I12)*.

*“…we can understand that TB-DM screening is a patient’s right to know the disease, on the other hand, related to the clinic we are worried about, referral services… because if we (many) refer, it will increase the number of referrals. We are a BPJS clinic, so the referral rate is limited to a maximum of 14% …”(I4)*.

In addition, we identified barriers associated with TB-DM screening decision-making in clinical management. This category involves determining the referral criteria and making the referral decision. The excerpt below illustrates barriers to decision-making in the clinical management of TB-DM screening.

*“… I am sometimes confused about the assessment of the screening instrument, what value is used as the basis for referrals, whether it is necessary to refer to the health center or the hospital, that is the obstacle…”(I11)*.

*“…when I read the weekly reports, I get the impression that some doctors are sometimes still confused, there are differences of opinion regarding the clinical suspicion of suspected TB patients, the duty officer is complex, sometimes they have to wait for me to be referred or not?“ (I7)*.

Furthermore, we also identified barriers associated with continuity of care. These barriers comprised suboptimal coordination between the government and the private sector, inadequate communication between public primary healthcare centers and private primary care clinics, financing for managing TB-DM patients, grouping DM patients, incomplete screening data, and regulatory issues with BPJS. The following quotes show the persistence of care-related hurdles.

*“…if the BPJS patient moves to the clinic from the Puskesmas, we do not have a previous history of diseases and treatment from there. There was once a patient who, after being identified, turned out to be TB-HIV, and we did not know about it beforehand. It was difficult for us to ask for this information, even though fellow health workers had to protect each other…”(I4)*.

*“…The funding from BPJS for the TB program is still being tug-of-war. They (BPJS) only want TB as a program. In terms of financing TB patients in hospitals, they are reluctant about it. They seem not to be in harmony with the government…” (I4)*.

This study found that clinic healthcare workers perceived TB screening interventions for diabetic patients as a relatively light additional burden. The burden is initially felt during the implementation phase. They believe they have been assigned additional tasks. However, after performing the service, they deemed it to be a simple task. TB-DM screening services can be incorporated into routine services and do not require a significant amount of time. They contend that TB screening increases their concern for TB disease. Excerpts from the interview reflect that implementing TB screening in DM patients is slightly burdensome.

*“I do not see a problem, as screening does not take up much time, and I prefer to converse with people.” (I5)*.

*“I don’t feel a heavy burden because the number of DM patients who are screened is still manageable, so this is quite easy to do.” (I7)*.

### Theme 3: strategies for improving TB-DM screening

According to the findings of the FGD, four screening procedure strategies and two screening necessities must be considered to implement TB screening in DM patients to achieve the intended results. These screening procedure strategies included TB-DM screening preparation, screening process, tiered screening, and follow-up screening.

We noticed that providing DM patients education prior to TB screening is a crucial first stage in ensuring the success of TB screening. This measure is anticipated to increase patient awareness of the significance of tuberculosis screening, thereby increasing their willingness to be screened. Interview excerpts illustrate strategies for screening preparation.

*“… so when DM patients come, we (nurses) provide education before the screening, sheets (screening instruments) are brought in when examined by doctors…” (I7)*.

*“… sometimes we distribute education through TB-DM leaflets to patients, or we put them in front (waiting room), so every visiting patient can read while waiting (before being screened)…” (I15)*.

Moreover, we revealed that care must be taken not to provoke the patient with TB screening queries directly during the screening procedure. As a precaution against the stigma associated with tuberculosis among patients, healthcare professionals chose not to explain the screening process explicitly. Electronic medical documents that included TB screening questions could be used to obfuscate TB tracing more directly and efficiently. Excerpts from interviews illustrate strategies for the vetting process.

*“…because (the screening instrument) is attached in the EMR (Electronic medical record), so it’s not too visible (by the patient)…we skim and ask questions like a normal history taking…” (I4)*.

*“…TB screening currently requires the cost of photocopy paper. One day, it might be even more economical if it can be included in the EMR…” (I7)*.

To obtain more reliable findings for TB screening, we obtained many clinics that used tiered screening. This tiered screening can involve the role of the patient registration unit, paramedics and doctors. Interview excerpts illustrate strategies for stratified screening.

*“…(for TB screening) sometimes (the one doing it) is the nurse, but if the patient (information) is difficult to understand, the doctor will explain again (screening)…“(I6)*.

*“.(screening) is carried out at the registration unit, followed by the nurse’s department, so we ask further (are there any symptoms) leading to TB. We know DM patients and direct them to the TB screening form, then give it to the doctor for further investigation…” (I14)*.

Patients with diabetes will undergo periodic repeat TB screening following the guidelines. Patients should be continually reminded to undergo screening at subsequent appointments. The reporting of TB screening results determined patient follow-up. The accompanying excerpts from informants illustrate strategies for follow-up screening.

*“… we usually say (to the patient) ‘sir, every month we will ask again for the TB screening, the patient answers ‘ok’…” (I7)*.

*“…In terms of reporting (screening results), there is currently no (routine) reporting, but for reporting TB diagnoses every month, we report to the Puskesmas (public primary health center)…” I14)*

Based on the FGD results, there were two TB-DM screening necessities to improve the screening implementation: internal and external needs. Internal needs included expanding the capacity of healthcare providers, upgrading buildings and infrastructure, and providing incentives. Interview snippets show the internal need for improving TB-DM testing.

*“…in my opinion, to obtain a more optimal screening, we should add more clinical staff. Because if the doctors change, sometimes the screening becomes less than optimal, not what we expected…” (I10).*

*“…the existing medical records, if possible, have a warning system to officers if a patient is present, a TB screening should be carried out. In addition, the TB screening form has been integrated into the electronic medical record, so we don’t have to look for scattered papers.” (I7)*.

While external requirements included the need to strengthen the community and collaborate with health institutions. Interview excerpts indicate the external need for enhancing TB-DM testing.

*“… so every patient is given a leaflet about TB to read and take home so that later when screened the patient will not be afraid…” (I7)*.

*“…to reduce the number of referrals to BPJS partner clinics, we hope that BPJS will separate the referral for suspected TB patients. Patients needing supporting examinations and specialist doctor consultation are not included in the referral rate…”(I4)*.

### Feasibility

In this study, the feasibility assessment refers to the extent to which the implementation of TB screening in DM patients can be successfully employed or carried out in private primary health facilities. Due to its practical criteria, we selected a feasibility instrument: The Feasibility of Intervention Measure (FIM) [24]. Higher scores indicate greater feasibility. Collecting data on this feasibility assessment used a survey at the organizational level in each clinic. The survey results are shown in Table 2. The majority of clinics reported that the implementation of TB-DM screening was practicable/feasible.

**Table 2:** Feasibility survey result

Indicator	Completely disagree (n%)	Disagree (n%)	Neither agree nor disagree (n%)	Agree (n%)	Completely agree (n%)
1. TB screening in DM patients seems implementable	0 (0%)	0 (0%)	0 (0%)	2 (40%)	3 (60%)
2. TB screening in DM patients seems possible	0 (0%)	0 (0%)	0 (0%)	1 (20%)	4 (80%)
3. TB screening in DM patients seems doable	0 (0%)	0 (0%)	0 (0%)	1 (20%)	4 (80%)
4. TB screening in DM patients seems easy to use	0 (0%)	0 (0%)	0 (0%)	1 (20%)	4 (80%)

## Discussion

This study is the first implementation research in Indonesia that specifically explores the implementation of TB screening among DM patients, especially in private primary care clinics. Our study’s results highlighted that TB screening among DM patients could be acceptable and feasible to be implemented in private primary care clinics. This study found that implementing TB-DM screening was attainable when properly implemented with consideration of enabling factors, hindering factors, and screening strategies to improve and enhance TB-DM screening. Furthermore, our study indicated that private primary care clinics required strategic planning before conducting TB-DM screening to achieve more optimal TB-DM screening.

Our study revealed TB-DM screening was found to be facilitated by an enthusiastic and cooperative patient, provider’s commitment, screening routines, and convenient SOPs, while fear and stigmatization of TB and COVID-19, fear of rising clinic referral rates, a suboptimal collaboration between government and private clinic, and regulatory issues with BPJS-*Kesehatan Indonesia* were identified as barriers to the successful implementation of TB-DM screening in private primary care clinics in Yogyakarta, Indonesia.

This study highlighted that the TB stigma continued to exist and hindered the implementation of TB-DM screening. This condition is consistent with the problem of TB control in Indonesia, where until now, TB stigma has had a negative impact on TB control, especially in reducing TB case detection [37]. Research from China, India, Ethiopia and Zambia also showed the same conclusion that TB-related stigma is still high and interferes with TB control [38–41]. DM patients may face additional stigma due to their underlying condition, which can further hinder TB screening and treatment uptake [42, 43]. Moreover, the co-occurrence of TB and DM can lead to diagnostic confusion, contributing to delays in TB diagnosis and treatment [44]. It can further exacerbate TB stigma among DM patients, leading to further delays in seeking healthcare. In some cases, DM patients may avoid TB screening altogether, leading to a missed opportunity for early diagnosis and treatment [45, 46].

This study revealed that the stigma of COVID-19 was also found to be a new obstacle in implementing TB-DM screening. The COVID-19 pandemic has negatively impacted global TB control, including TB screening programs. Several reports found a decrease in TB testing during COVID-19 (2020) compared to the previous period (2019) [47]. Implementation of TB-DM screening in the context of the ongoing COVID-19 pandemic presents a real challenge. The COVID-19 pandemic has triggered stigmatization and discriminatory behavior towards people who have or may have, COVID-19 [48]. The symptoms of TB are similar to those of COVID-19, such as coughing, fever, and shortness of breath. As a result, COVID-19 stigma has affected the efforts to screen and diagnose TB. People with TB symptoms may hesitate to seek medical attention, fearing being stigmatized and discriminated against [49]. Furthermore, the fear of contracting COVID-19 may also deter individuals from seeking TB screening services due to the fear of contracting COVID-19. Therefore, COVID-19 may reduce TB screening, resulting in delayed TB diagnosis and care [50].

Another important finding was that implementing TB screening in DM patients in private primary care clinics poses a dilemma. On the one hand, clinics must be able to increase TB case detection, while on the other, the number of patients referred for TB tracing influences the number of clinic referrals. This issue might be explained by the fact that private clinics are subject to a quota imposed by BPJS *Kesehatan Indonesia*, which specifies that no more than 15% of BPJS patients may be referred in a given month [51]. This regulatory issue was a cautionary consideration for private clinics referring patients suspected of having TB based on TB-DM screening.

Our study revealed a suboptimal collaboration between private primary healthcare clinics and the government. The absence of effective communication and coordination between the private sector and the government might be one of the reasons. Mailu et al. (2019) noted that poor communication between the two entities could lead to duplication of efforts, inefficient use of resources, and poor TB control outcomes [52]. Additionally, the lack of a formal mechanism for collaboration between the government and the private sector is another barrier to effective collaboration [53]. Another issue is the lack of trust between the private and government sectors. According to Ananthakrishnan et al. (2019), private providers may perceive the government’s involvement in their practice as intrusive, leading to resistance to collaboration [54]. Additionally, the government may not trust private providers to report accurate TB data, leading to a lack of confidence in their ability to contribute to TB control efforts [55]. Moreover, the financial incentives for private providers may be misaligned with the public health goals of TB control. Private providers may be more focused on generating revenue from patients than on contributing to public health outcomes, leading to suboptimal collaboration with the government [56]. The lack of financial incentives for private providers to report TB cases to the government is also a significant issue [57].

The results of our study from the feasibility framework revealed that most clinics stated that TB-DM screening could be carried out. Most feasibility survey data reports agree and strongly agree that TB-DM screening can be implemented in the context of private primary health care in Indonesia. The feasibility of TB screening among DM patients has been investigated in many studies. Several studies conducted in India demonstrated that screening for TB among DM patients was feasible in India, where the DM epidemic continues to rise [46, 58]. Similarly, a study conducted in Bangladesh indicated that TB screening among DM patients was also feasible [59]. It aligns with a pilot project study of TB-DM screening among patients visiting clinics in China, showing it was also feasible. The study recommended optimizing the application of TB-DM screening to identify people with diabetes who are at a higher risk for TB [60]. A study conducted in South Nigeria found that the yield of TB cases among DM patients was effective and identified a high proportion of TB cases [61]. Another study in Pakistan revealed that TB screening among DM patients was feasible and effective in detecting active TB cases [62].

However, other studies have shown that TB screening among DM patients might not be feasible. A study conducted in North India found screening for TB among DM patients was not implemented, despite documents indicating that it had been [63]. Another study in India showed that they failed to yield any active TB cases using a WHO-recommended questionnaire among people with DM [64]. Moroever, a study conducted in Tanzania found that DM patients were less likely to present with TB symptoms, which may limit the effectiveness of TB screening [65]. A study in Sri Lanka demonstrated that active screening for pulmonary TB among DM patients in clinics was non-effective at improving TB case findings [66].

Despite these various findings, several studies have suggested that TB screening among DM patients is feasible and effective [45, 64, 66, 67]. Overall, the feasibility of TB screening among DM patients appears to be context-specific and may depend on factors such as the prevalence of TB and DM in the population, the availability of screening tools, and the cost-effectiveness of screening strategies. Therefore, it is important to consider these factors when designing TB screening programs for DM patients.

Incorporating TB screening protocols for diabetic patients into daily clinical practice is a possibility that is both promising and harmonious, as shown by the positive findings of this study. By demonstrating the acceptance of healthcare providers to conduct these screenings, we can anticipate more proactive and comprehensive care for diabetes patients, ultimately leading to earlier detection and treatment of TB cases in this vulnerable population. In addition, the implementation’s practicality is highlighted by the feasibility aspect. Recognizing that healthcare providers can conduct TB-DM screening effectively in private primary care clinics demonstrates the sustainability and scalability of this approach. The findings of this study have the potential to not only improve patient outcomes but also serve as a model for similar initiatives in a broad range of healthcare settings, thereby making a substantial contribution to improving public health and healthcare delivery systems.

### Study limitations

Our study has limitations that must be considered to avoid overgeneralization. First, our study was conducted in some faith-affiliate private primary health care clinics in Yogyakarta, Indonesia. Therefore, the findings must be interpreted judiciously in different settings. Second, several clinics participating in the study had varying numbers of DM patient visits. As a result, experiences may differ among healthcare workers. Third, the implementation of TB-DM screening was carried out during the COVID-19 pandemic. The stigma of COVID-19 has become a new obstacle in the research process. The existence of a social restriction policy makes data collection techniques becomes limited.

## Conclusions

The findings indicated that our study provided evidence that implementing TB screening in DM patients in private primary care clinics can be acceptable and feasible. Enabling factors, hindering factors, and strategies for improving TB-DM screening must all be considered for successful implementation.

## Data Availability

The dataset for this study is not publicly available du to stipulations of ethical approval but can be made availabe from the corrresponding author upon reasonable request.

## List of abbreviations

TB: Tuberculosis
DM: Diabetes Mellitus
WHO: World Health Organization
Mtb: Mycobacterium tuberculosis
COVID-19: Corona Virus Disease-19
CHCs: Community health centers
NTPs: National Tuberculosis Programs
PPCs: Private Primary Cares
FGD: Focus Group Discussion
BPJS: *Badan Penyelenggara Jaminan Sosial* (Social Security Organizing Agency)
TFA: Theoretical Framework of Acceptability
SOP: Standard Operating Procedure
FIM: Feasibility of Intervention Measure
AIM: Acceptability of Intervention Measure
IAM: Intervention Appropriateness Measure
